# RELATIONSHIP OF AGING EXPECTATIONS, GOAL CONGRUENCE, AND ACTIVITY TO MUSCLE FUNCTION IN OLDER ADULTS

**DOI:** 10.1093/geroni/igz038.1923

**Published:** 2019-11-08

**Authors:** Murad H Taani, Christina Sima, Immaculate Apchemengich, Andrew Kaplan, Michael Fendrich, Rachel Schiffman, Scott Strath

**Affiliations:** 1 University of Wisconsin Milwaukee, Milwaukee, Wisconsin, United States; 2 University of Connecticut, Hartford, Connecticut, United States

## Abstract

Poor muscle function is a major source of disability among older adults and leads to negative health outcomes including falls and fractures, exacerbating healthcare cost. This study was undertaken to understand: a) the characteristics of muscle function; and b) the relationship of self-management process variables (expectations regarding aging, goal congruence, and self-efficacy for physical activity) and physical activity self-management behavior to muscle function in a sample of older adults (N = 65) 75-93 years of age living in Continuing Care Retirement Communities. Using a descriptive correlational design, muscle function was measured with the Short Physical Performance Battery (SPPB) test and physical activity level with ActiGraph GT3X. Questionnaires included Expectations Regarding Aging and goal congruence scales and Physical Activity Assessment Inventory to assess self-efficacy. Pain was assessed by the PROMIS Pain Intensity 3a. Most participants (77%) performed poorly on the SPPB test. Controlling for pain, expectations regarding aging, goal congruence, self-efficacy and physical activity explained 46% of the variance in SPPB score. The model demonstrated that self-efficacy and light-intensity physical activity significantly explained 24.6 % of the variance in SPPB score; suggesting that low self-efficacy and decreased levels of light-intensity physical activity were significant predictors of low SPPB score. The findings demonstrate the need for more research documenting the underlying processes and risk factors for reduced muscle function. The potential benefits of this approach provide a basis for designing interventions to improve muscle function and delay the transfer to more restrictive living environments.

